# Occurrence of *Vibrio* Pathotypes in the Final Effluents of Five Wastewater Treatment Plants in Amathole and Chris Hani District Municipalities in South Africa

**DOI:** 10.3390/ijerph110807755

**Published:** 2014-08-04

**Authors:** Vuyokazi Nongogo, Anthony I. Okoh

**Affiliations:** Applied and Environmental Microbiology Research Group (AEMREG), Department of Biochemistry and Microbiology, University of Fort Hare, Alice 5700, South Africa; E-Mail: AOkoh@ufh.ac.za

**Keywords:** wastewater treatment plant, effluents, *Vibrio* pathotypes

## Abstract

We assessed the occurrence of *Vibrio* pathogens in the final effluents of five wastewater treatment plants (WWTPs) located in Amathole and Chris Hani District Municipalities in South Africa over a 12 months period between September 2012 and August 2013 using standard membrane filtration technique followed by cultivation on thiosulphate citrate-bile salts-sucrose (TCBS) agar. The identities of the presumptive *Vibrio* isolates were confirmed using polymerase chain reaction (PCR) including delineation into *V. parahaemolyticus*, *V. vulnificus* and *V. fluvialis* pathotypes. The counts of *Vibrio* spp. varied with months in all the study sites and ranged in the order of 10^1^ and 10^4^ CFU/100mL. *Vibrio* distribution also showed seasonality with high counts being obtained in autumn and spring (*p* < 0.05). Prevalence of *Vibrio* spp. among the five WWTPs also differed significantly (*p* < 0.05). Of the 300 isolates that were confirmed as belonging to the *Vibrio* genus, 29% (86) were *V. fluvialis*, 28% (84) were *V. vulnificus* and 12% (35) were *V. parahaemolyticus*. The isolation of *Vibrio* pathogens from the final effluent suggests that this pathogen is in circulation in some pockets of the population and that the WWTPs under study do not efficiently remove bacterial pathogens from the wastewater and consequently are threats to public health.

## 1. Introduction

Wastewater production is a common phenomenon worldwide and regulatory imperatives demands that wastewater be treated before discharge into the environment [1]. Unfortunately, many wastewater treatment plants still discharge significant amounts of fecal coliforms and pathogenic microorganisms which impair the quality of water in the receiving watersheds [2]. The impaired quality of these final effluents is usually brought about by the poor operational state and inadequate maintenance of most of these municipalities’ sewage treatment works resulting in production of effluents of poor quality [3] thus impacting negatively on the receiving watersheds. Wastewater final effluents therefore serve as reservoirs of many enteric pathogens [4] especially also capitalizing on their enhanced strategies to survive wastewater treatment processes and reduced susceptibilities to disinfectants [5]. Of these enteric pathogens, the *Vibrio* genus has been one of the major pathogens known to cause outbreaks worldwide, but mostly known for causing cholera [6]. The genus *Vibrio* is a member of the family *Vibrionaeceae* which includes opportunistic pathogens of humans and animals [7]. They are marine in origin, and commonly associated with aquatic living species [8]. Although they can be pathogens for humans and aquatic animals, their role in the marine environment has been shown to include biodegradation, nutrient regeneration and biogeochemical cycling [9]. Their adaptability to adverse conditions has promoted wide distribution of vibrios in effluent environments associated with domestic sewage [10]. Previous research has focused mostly on *Vibrio cholera* in water because of the severity of the disease it causes [11], but over the last decade, several studies have involved relatively minor *Vibrio* species of medical interest [7], some of which are described as emerging pathogens able to cause mild to severe human diseases [12].

Several species of *Vibrio* are pathogens, including *V. parahaemolyticus*, *V. fluvialis* and *V. vulnificus* which is common in warm seawater and thrives in water temperatures greater than 20 °C [13]. This pathogen is directly associated with pollution or fecal waste and in most cases, causes disease in individuals who eat contaminated seafood (usually raw or undercooked oysters) or have an open wound that is exposed to seawater [14]. The result of exposure to *V.* *vulnificus* usually results in wound infections, gastroenteritis, or primary septicaemia [15]. *Vibrio parahaemolyticus* occupies a variety of niches and is a common bacterium in marine and estuarine environments [16]. Though this organism is recognized as a major worldwide cause of gastroenteritis, particularly in areas of the world where seafood consumption is high [17], an interesting study by Tunung *et al.* [18] has reported prevalence of *V. parahaemolyticus* also in raw vegetables from retail shops. *Vibrio fluviali*s is a halophilic *Vibrio* species that has been associated with sporadic outbreaks of diarrhoea worldwide [19,20,21], and is clinically very similar to cholera. *V. fluvialis* can also pose a significant economic threat to aquaculture since it is pathogenic to cultured fish and lobsters [22]. Infections by *V. fluvialis* are generally common in infants, children, and young adults [23].

Studies concerning *Vibrio* pathogens have focused mainly on seafood and the marine environment. There is a dearth of information on the incidence of *Vibrio* species in wastewater effluents worldwide and to the best of our knowledge, only studies from our group [24,25] have reported on wastewater effluent vibriology in South Africa, albeit in two facilities, and the question of how widespread this phenomenon is in the Eastern Cape Province of South Africa became a motivation for this current study. In this paper, we evaluate the occurrence of *Vibrio* pathotypes in the final effluents of five wastewater treatment plants in the Amathole and Chris Hani district municipalities in the Eastern Cape Province as part of our larger study on wastewater effluents vibriology in South Africa.

## 2. Experimental Section

### 2.1. Description of Study Site

The 5 WWTPs are located in Amathole (Plants E, M and R) and Chris Hani (Plants Q and W) district municipalities in the Eastern Cape Province of South Africa which is one of the poorest and second largest provinces in South Africa, mainly comprised of rural settlements with little or no adequate sanitary facilities [26]. Plant M and Plant W use biofilter treatment technology while Plant R, Plant E and Plant Q use activated sludge systems. The operational characteristics of the plants are as articulated in Table 1.

**Table 1:** Some characteristics of the WWTPs.

WWTP	Amathole D.M WWTPs			Chris Hani D.M WWTPs	
	Plant M	Plant R	Plant E	Plant W	Plant Q
Technology	Biofilters, anaerobic digestion and sludge drying beds	Activated sludge and sludge lagoons	Activated sludge and marine outfall	Biofilters, sludge composting	Biofilters, anaerobic digestion
Design Capacity (ML/d)	24	2.5	40	4.99	NI *
Operational % in relation to Design Capacity	43.8%	44%	85.5%	50.1%	NI *

NI * denotes that No Information was provided on Plant Q from the DWAF Greendrop report of 2012.

### 2.2. Sample Collection

Wastewater final effluent samples were collected aseptically from the final effluents using sterile 1000 mL glass bottles containing 1.7 mL of 1% sodium thiosulfate for de-chlorination. Samples were transported on ice to the laboratory of the Applied and Environmental Microbiology Research Group (AEMREG) at the University of Fort Hare for analysis within 6 h of collection.

### 2.3. Enumeration and Isolation of Presumptive Vibrio Species

*Vibrio* bacteria count was done using the membrane filtration method. Briefly, 100 mL of appropriately diluted effluent samples was filtered through a 0.45 μm size membrane filters under vacuum. The membrane filter was then transferred onto thiosulphate citrate bile salts sucrose (TCBS) agar plates and incubated at 37 °C for up to 48 h. At the end of the incubation period, typical yellow and green colonies were counted as presumptive *Vibrio* species and expressed as colony forming units per 100 mL (CFU/100 mL). Five to 10 isolated colonies per plate were then randomly picked and subsequently subcultured on sterile TCBS agar plates for purity. Pure isolates were then plated on nutrient agar plants, incubated overnight as before and from there glycerol stocks (20%) were prepared and stored at −80 °C for further analysis.

### 2.4. Molecular Confirmation of Pathogenic Vibrio Species

Variable regions around positions of 700 and 1325 within the 16S rRNA gene were used as target sequences to confirm the identities of the presumptive *Vibrio* isolates to the genus level using specific primers in the polymerase chain reaction (PCR) assay [27]. PCR was also done to further delineate the confirmed *Vibrio* isolates into *V. fluvialis*, *V. vulnificus* and *V.* *parahaemolyticus* species using species-specific primers targeting the different regions of the *toxR* and *hsp*60 gene as presented in Table 2. To isolate the genomic DNA the method of Maugeri *et al.* [28] was followed. Single colonies of presumptive *Vibrio* grown overnight at 37 °C on nutrient agar plates were picked, suspended in 200 μL of sterile distilled water and the cells lysed using AccuBlock (Digital dry bath, Labnet) for 15 min at 100 °C. The cell debris was removed by centrifugation at 11000 × *g* for 2 min using a MiniSpin micro centrifuge. The cell lysates (5 μL) was used as template in the PCR assays immediately after extraction. The thermal cycling profile was as follows: a single round of enzyme activation for 15 min at 93 °C followed by 35 cycles at 92 °C for 40 s, 57 °C for 1 min and 72 °C for 1.5 min and final extension at 72 °C for 7 min.

**Table 2:** Sets of primers used for identification and pathotyping of *Vibrio* species.

Target Species	Primers	Sequences (5’3’)	Target Gene	Amplicon Size (bp)	Reference
All *Vibrio* spp.	V. 16S-700F V. 16s-1325R	CGG TGA AAT GCG TAG AGA T TTA CTA GCG ATT CCG AGT TC	16SrRNA	663	[27]
*V. parahaemolyticus*	Vp.toxR R Vp.toxR F	GTC TTC TGA CGC AAT CGT TG ATA CGA GTG GTT GCT GTC ATG	*toxR*	368	[29]
*V. vulnificus*	Vv. hsp-326F Vv. hsp-697R	GTC TTA AAG CGG TTG CTG C CGC TTC AAG TGC TGG TAG AAG	*hsp*60	410	[30]
*V. fluvialis*	Vf- toxR F Vf- toxR R	GAC CAG GGC TTT GAG GTG GAC AGG ATA CGG CAC TTG AGT AAG ACT C	*toxR*	217	[31]

## 3. Results and Discussion

*Vibrio* densities during the study period ranged between 1–1.48 × 10^4^ CFU/100 mL. High densities of 1.28 × 10^4^ CFU/100 mL and 1.48 × 10^4^ CFU/100 mL were obtained for the months of November 2012 and May 2013 at Plant E and Plant Q WWTPs, respectively as shown in Table 3.

**Table 3:** Occurrence of *Vibrio* spp. in selected WWTPs from the Amathole and Chris District Municipalities.

*Vibrio* spp. (CFU/100 mL)												
WWTP	SEPT ’12	OCT ’12	NOV ’12	DEC ‘12	JAN’13	FEB’13	MAR’13	APR’13	MAY’13	JUNE ’13	JULY’13	AUG’13
PLANT M	9.2 × 10^2^	4.8 × 10^1^	6.2 × 10^2^	1.6 × 10^2^	1.0 × 10^2^	2.6 × 10^1^	5.2 × 10^2^	6.0 × 10^1^	1.3 × 10^1^	1.4 × 10^1^	9.5 × 10^1^	1.3 × 10^1^
PLANT R	<1	<1	1.3 × 10^0^	2 × 10^0^	1 × 10^0^	<1	<1	8.7 × 10^0^	9 × 10°	<1	5 × 10^0^	<1
PLANT E	3.6 × 10^1^	8.5 × 10^2^	1.28 × 10^4^	6.1 × 10^2^	5.2 × 10^2^	3.5 × 10^1^	6.2 × 10^1^	7.7 × 10^3^	6.0 × 10^1^	3.5 × 10^2^	4.5 × 10^3^	1.29 × 10^3^
PLANT W	N/S	1.14 × 10^2^	1.61 × 10^2^	1.8 × 10^2^	4.0 × 10^1^	3.9 × 10^1^	7.2 × 10^1^	1.6 × 10^1^	2.1 × 10^1^	< 1	1.6 × 10^1^	7 × 10^0^
PLANT Q	6.8 × 10^1^	3.4 × 10^2^	2.26 × 10^3^	1.48 × 10^3^	9.6 × 10^1^	8.3 × 10^3^	1.24 × 10^3^	5.8 × 10^1^	1.48 × 10^4^	2.5 × 10^1^	1 × 10^0^	<1

NS = not sampled; CFU = colony forming units; WWTP = wastewater treatment plant; <= less than.

Plant Q was noted to have challenges with the pipeline system necessitating upgrading of the plant between the months of September 2012–May 2013. As shown in Table 3, the highest counts of presumptive *Vibrio* species were obtained in the months when maintenance work was ongoing. However, after the refurbishment of Plant Q, the plant had major improvements and experienced counts as low as 1 CFU/100 mL in July 2013 and <1 CFU/100 mL in August 2013. One other possibility of the reduction in the *Vibrio* counts could be the winter season which normally starts from June to August and characterized by low temperatures in South Africa. Similar studies by Igbinosa *et al.* [24] showed that the abundance of *Vibrio* species in the final effluents was linked to temperature, while its relationship to salinity is less clear. The remaining WWTPs, *i.e.*, Plant M and Plant W, were also characterized with high *Vibrio* counts in the order of 10^2^ CFU/100 mL. The *Vibrio* counts in Plant M did not follow any defined pattern and fluctuated throughout the sampling period and suspected to be related to irregular and inadequate chlorine disinfectant dosing regimens. Also, the presence of a kraal nearby suggest the possibility of post-treatment contamination of the final effluent tank by run-offs from the kraal as suggested by the report of Uddin *et al.* [32] on the prevalence of *Vibrio* from cow dung and excreta of poultry samples.

**Figure 1:**
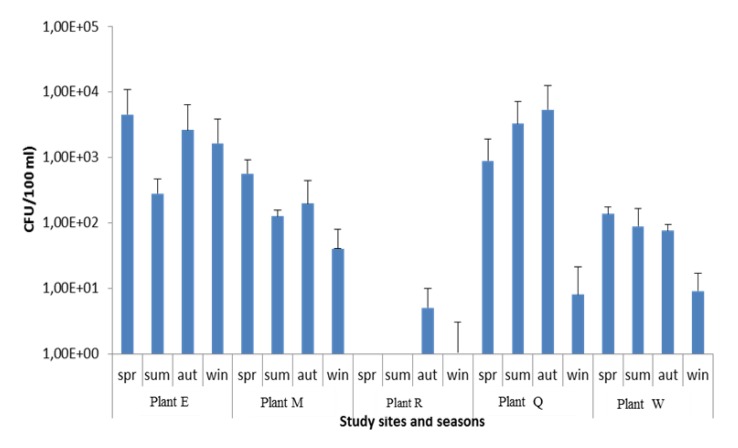
Seasonal distribution of *Vibrio* spp. in selected wastewater treatment plants.

With respect to season, the *Vibrio* counts (Figure 1) varied significantly (*p* ≤ 0.05), and was highest in autumn for Plant Q (5.4 × 10^3^ CFU/100 mL) and in spring for Plant E (4.6 × 10^3^ CFU/100 mL). However, Plant R recorded the lowest *Vibrio* counts throughout the seasons. There was a similar trend in Plants E, M and W where the highest mean counts were obtained during spring and the lowest mean counts in winter. These findings corroborate the observations of Lin and Schwarz [33] who reported that *V. vulnificus* was not detected during the winter months but abundantly isolated in the spring months. Maugeri *et al.* [34] also has confirmed that the distribution of pathogenic *Vibrio spp.* in aquatic environments is greatly influenced by temperature. Plant R and Plant Q had their highest mean *Vibrio* densities in autumn. The lowest mean counts were obtained in winter for Plant Q and in spring and summer for Plant R.

**Figure 2:**
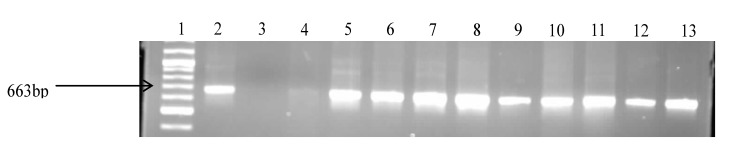
Gel electrophoresis of the PCR products of some of the confirmed *Vibrio* spp. Lane 1: Gene ruler (100 bp); Lane 2: Positive (+ve) control *V. fluvialis*; Lane 3: Negative control; Lane 4–13 (Samples).

Molecular confirmation of the presumptive *Vibrio* isolates resulted in the confirmation of 300 isolates as belonging to the *Vibrio* genus as shown in Figure 2.

**Figure 3:**
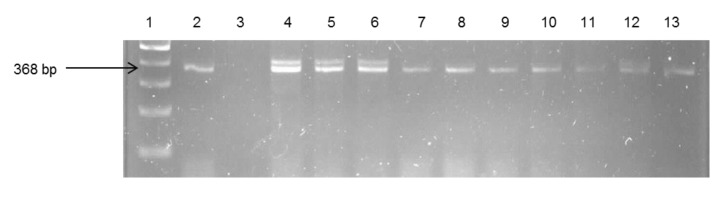
Gel electrophoresis of PCR products of some of the confirmed *V. parahaemolyticus*. Lane 1: Gene ruler (100 bp); Lane 2: Positive control (*V. parahaemolyticus* DSM 11058); Lane 3 (Negative control); Lane 4–13 (Samples)

**Figure 4:**
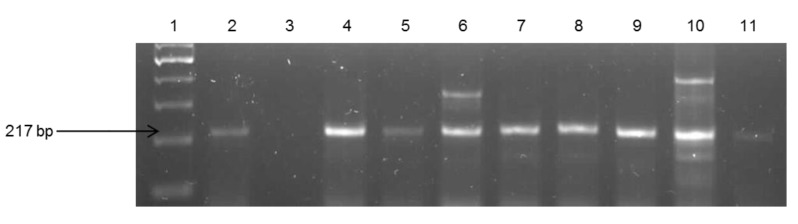
Gel electrophoresis of PCR products of some of the confirmed *V. fluvialis*. Lane 1: Gene ruler (100 bp); Lane 2: Positive control (*V. fluvialis* DSM 19283*)*; Lane 3 (Negative control); Lane 4–11 (Samples).

**Figure 5:**
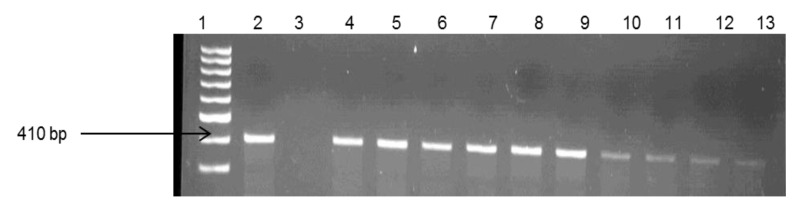
Gel electrophoresis of PCR products of some of the confirmed *V.* *vulnificus*. Lane 1: Gene ruler (100 bp); Lane 2: Positive (+ve) control *V. vulnificus* (410 bp); Lane 3 (Negative control); Lane 4–13 (Samples).

About 29% of the isolates were found to be *V. fluvialis*, while 28% were *V. vulnificus* and 11.6% were *V. parahaemolyticus.* The remaining isolates (31.8%) belonged to other species that were not assayed for in this study. The identities of *V. fluvialis* and *V. parahaemolyticus* were confirmed by use of species-specific primers targeting the different regions of the *toxR* gene. Identification of the confirmed isolates into respective *Vibrio* pathotypes reveals the presence of potentially pathogenic strains for humans and animals. Gel electrophoresis of the species delineation PCRs are as shown in Figure 3, Figure 4 and Figure 5.

It is known that among vibrios, these three species can adapt themselves to adverse conditions e.g., organic matter limited environments by means of survival strategies such as adhering to different substrata [28,35] hence survival of these pathogenic vibrios in wastewater treatment plants is possible. The most prevalent species detected was *V. fluvialis* followed by *V. vulnificus* and *V.* *parahaemolyticus*. Previous reports have articulated the isolation of *Vibrio* species from different niches and geographical regions. In marine environments of Italy Gugliandolo *et al.* [35] found *V. vulnificus* as a dominant species, while Sousa *et al.* [36] isolated *V. parahaemolyticus* and *V. cholera* in oysters collected in Brazil. Also Tuning *et al*. [18] has highlighted the prevalence of *V. parahaemolyticus* in raw vegetables.

It has also been reported that human activity can greatly enhance the global transport of marine species [37] including pathogenic strains and may have contributed to the isolation of *Vibrio* in the final effluents. Findings of Kelly and Stroh [38] from Pacific Northwest showed that oysters are the main source of *V. fluvialis* and other vibrios especially during warmer seasons. The dangers which come with the abundance of *V.* *fluvialis*is the production of an enterotoxin known to cause a serious infection, as its clinical symptoms of gastroenteritis are very similar to those caused by *V. cholera* O1 and non-O1 strains [19]. According to a recent study Liang *et al.* [39], *Vibrio fluvialis* has been considered to be an emerging food borne pathogen and has become a high human public health hazard all over the world, especially in coastal areas of developing countries and regions with poor sanitation. Isolation of *V. fluvialis* in large numbers can pose a significant economic threat for aquaculture in areas where seafood consumption is high, making a cycle back to faecal waste and final effluents [22]. Similarly, both *V. vulnificus* and *V. parahaemolyticus* are also food borne pathogens which are associated with raw seafood causing 3 major syndromes of clinical illness, *i.e.*, gastroenteritis, wound infections, and septicaemia [40]. *V.* *parahaemolyticus* has been often isolated from seafood, including shrimp, in markets in South East Asian countries [41] and previous studies at markets in China have shown *V.* *vulnificus* as dominant in cultured shrimps [42,43]. 

## 4. Conclusions

In this study, the presence of *Vibrio* pathogens mainly *V. fluvialis*, *V.* *vulnificus* and *V. parahaemolyticus* in the final effluents of WWTPs suggest that wastewater effluents are important reservoirs of *Vibrio* pathotypes and potential source of the same in the watershed. The presence of these pathogens in high densities also suggest the inefficiency of the treatment plants to adequately remove microbial pathogens from wastewater, and as such, constitute a threat to public and environmental health. The need for regular monitoring of the treatment works to ensure compliance to set guidelines becomes imperative and here recommended.
